# The Effect of K_*ATP*_ Channel Blocker Glibenclamide on CGRP-Induced Headache and Hemodynamic in Healthy Volunteers

**DOI:** 10.3389/fphys.2021.652136

**Published:** 2021-06-11

**Authors:** Hande Coskun, Fatima Azzahra Elbahi, Mohammad Al-Mahdi Al-Karagholi, Hashmat Ghanizada, Majid Sheykhzade, Messoud Ashina

**Affiliations:** ^1^Danish Headache Center, Department of Neurology, Rigshospitalet Glostrup, Faculty of Health and Medical Sciences, University of Copenhagen, Copenhagen, Denmark; ^2^Department of Drug Design and Pharmacology, Faculty of Health and Medical Sciences, University of Copenhagen, Copenhagen, Denmark; ^3^Danish Headache Knowledge Center, Rigshospitalet Glostrup, Glostrup, Denmark

**Keywords:** humans, migraine, glyburide, calcitonin-gene related peptide, cranial arteries

## Abstract

**Background:**

Calcitonin gene-related peptide (CGRP) dilates cranial arteries and triggers headache. The CGRP signaling pathway is partly dependent on activation of ATP-sensitive potassium (K_*ATP*_) channels. Here, we investigated the effect of the K_*ATP*_ channel blocker glibenclamide on CGRP-induced headache and vascular changes in healthy volunteers.

**Methods:**

In a randomized, double-blind, placebo-controlled, cross-over study, 20 healthy volunteers aged 18–27 years were randomly allocated to receive an intravenous infusion of 1.5 μg/min CGRP after oral pretreatment with glibenclamide (glibenclamide-CGRP day) or placebo (placebo-CGRP day). The primary endpoints were the difference in incidence of headache and the difference in area under the curve (AUC) for headache intensity scores (0–14 h) between glibenclamide and placebo. The secondary endpoints were the difference in AUC for middle cerebral artery blood flow velocity (V_*MCA*_), superficial temporal artery (STA) and radial artery (RA) diameter, facial flushing, heart rate (HR) and mean arterial blood pressure (MAP) (0–4 h) between glibenclamide and placebo.

**Results:**

We found no significant difference in the incidence of headache between glibenclamide-CGRP day (14/20, 70%) and placebo-CGRP day (19/20, 95%) (*P* = 0.06). The AUC for headache intensity, V_*MCA*_, STA, RA, facial skin blood flow, HR, and MAP did not differ between glibenclamide-CGRP day compared to placebo-CGRP day (*P >* 0.05).

**Conclusion:**

Pretreatment with a non-selective K_*ATP*_ channel inhibitor glibenclamide did not attenuate CGRP-induced headache and hemodynamic changes in healthy volunteers. We suggest that CGRP-induced responses could be mediated via activation of specific isoforms of sulfonylurea receptor subunits of K_*ATP*_ channel.

## Introduction

Calcitonin gene-related peptide (CGRP) is a potent vasodilator of cranial arteries (Brain et al., 1985; Falkenberg et al., 2020) and intravenous infusion of CGRP causes headache in healthy volunteers (Petersen et al., 2005b; Falkenberg et al., 2020) and migraine attacks in migraine patients (Lassen et al., 2002; Hansen et al., 2010; Ashina, 2020; Iljazi et al., 2020; Ashina et al., 2021). The molecular mechanisms by which CGRP mediates head pain are still unclear (Ashina et al., 2019). Recent studies suggested K_*ATP*_ channels as downstream effectors in the CGRP signaling pathway (Nelson et al., 1990; Kitazono et al., 1993; Quayle et al., 1994; Kleppisch and Nelson, 1995). K_*ATP*_ channels are expressed in neurons, vascular endothelium, and smooth muscle cells. These channels are involved in diverse physiological processes including insulin secretion, regulation of vascular tone, and protecting against metabolic stress (Al-Karagholi et al., 2017, 2019a, 2020b). In human provocation studies, K_*ATP*_ channel opener levcromakalim induces headache and migraine (Al-Karagholi et al., 2019b, c, 2020a, 2021a).

The widely used antidiabetic drug, glibenclamide, is a non-selective K_*ATP*_ channel inhibitor that belongs to the second-generation of sulfonylurea (Luzi and Pozza, 1997; Hibino et al., 2010). Preclinical evidence has demonstrated that glibenclamide attenuates CGRP-induced cranial artery dilation (Gozalov et al., 2005, 2008; Al-karagholi et al., 2019d) and trigeminal pain transmission (Christensen et al., 2020). In this study, we hypothesized that glibenclamide would inhibit CGRP-induced headache and vascular changes. To investigate this, we conducted a double-blind, randomized, placebo-controlled, crossover study in healthy volunteers.

## Materials and Methods

Twenty healthy volunteers were recruited through the Danish website^1^. Written informed consent was obtained from all participants after detailed oral and written study information. The female participants were required to have sufficient contraception [contraceptive pill or intrauterine device/system (IUD/IUS)]. Exclusion criteria were: (1) history of cardiovascular or cerebrovascular disease, diabetes mellitus, or hypercholesterolemia, (2) electrocardiogram (ECG) changes indicative of myocardial ischemia or hypertrophy, (3) hypertension at baseline on an experimental day (defined as a systolic blood pressure above 150 mmHg or a diastolic blood pressure above 100 mmHg), (4) current pregnancy or breastfeeding, (5) daily intake of medication (except oral contraceptives), (6) daily smoking within last 5 years, (7) first-degree relatives with a history of diabetes mellitus, and (8) history of any primary headache disorders (except episodic tension-type headache for <2 days per month during the last year) or first-degree family members with migraine as defined by the third International Classification of Headache Disorders (ICHD) (Vincent and Wang, 2018). A full medical examination and ECG were performed on the day of recruitment.

The study was approved by the Ethics Committee of the Capital Region of Denmark (H-19065735) and the Danish Data Protection Agency, and was conducted according to the Declaration of Helsinki of 1964, with later revisions. The study was also registered at ClinicalTrials.gov (NCT04231617).

### Experimental Design and Randomization

**FIGURE 1:**
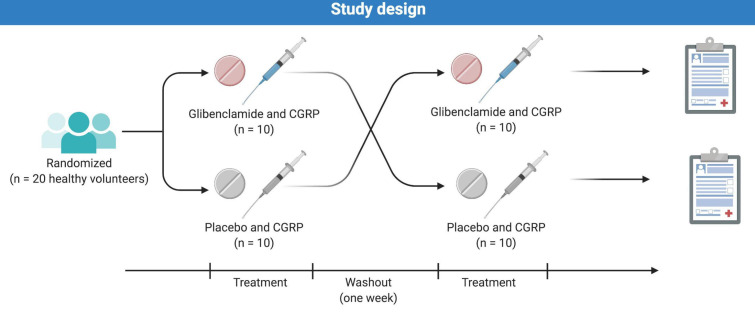
Twenty healthy volunteers (11 women and 9 men) were randomly allocated to receive oral glibenclamide or placebo followed by a 20 min CGRP-infusion. The two study days were separated by at least 7 days to ensure proper wash-out.

In a double-blind, placebo-controlled, crossover design, the participants were in a balanced order randomly allocated to receive an intravenous infusion of 1.5 μg/min CGRP over 20 min, 2 h after oral pretreatment with either 10 mg glibenclamide (Hexaglucon, Sandoz) or placebo (calcium supplement tablet) on 2 days separated by at least 1 week (Figure 1). Preparation of the study drug was performed by the Capital Region Central Pharmacy. Randomization and drug allocation were carried out by personal unrelated to the study to ensure study staff, participants and investigator remained blinded. Investigators had no access to the randomization code until the study was completed.

**FIGURE 2:**
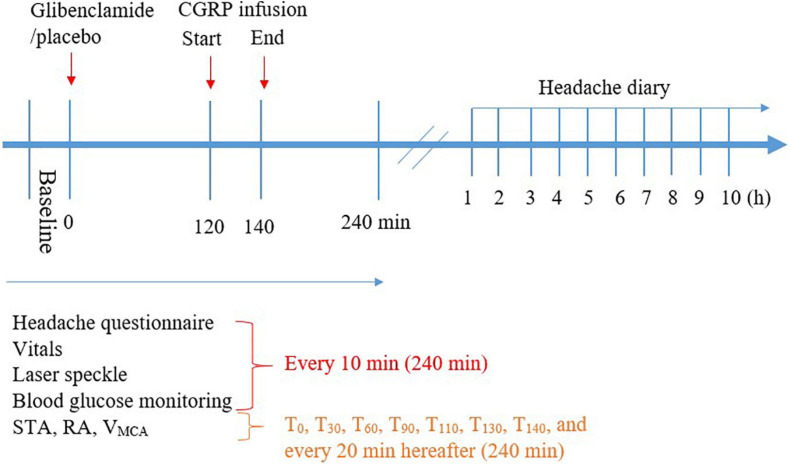
Timeline of the procedure during hospital phase (0–240 min) and post-hospital phase (1–10 h).

Participants arrived non-fasting at the clinic between 8:00 and 9:00 AM on each study day. The participants were placed in the supine position and a venous catheter was inserted into the left and right antecubital vein for drug (CGRP) and 20% glucose infusion. Then, participants rested for at least 30 min before baseline measurements of vital signs were performed. The infusion started using a time and volume-controlled infusion pump. Vital signs including mean arterial blood pressure (MAP), heart rate (HR), respiratory rate, blood oxygen saturation and nasal end-tidal CO_2_ tension were continuously monitored and recorded every 10 min (ProPac Encore; Welch Allyn Protocol) (Figure 2). Room temperature was continuously monitored and recorded every 5 min. Facial flushing was measured by speckle contrast imager (moorFLPI; Full Laser Perfusion Imager). The contrast imager was placed 30 cm perpendicularly above the face of the participants and measured the skin blood flow automatically every 5 s as previously described (Ghanizada et al., 2020a). Middle cerebral artery blood flow velocity (V_*MCA*_), left superficial temporal artery (STA) diameter, left radial artery (RA) diameter, and end-tidal partial pressure of CO_2_ (PetCO_2_) were recorded as previously described (Petersen et al., 2005a).

MA and HG evaluated eligibility, obtained informed consent, and enrolled the participants. Experiments were carried out at the Danish Headache Center, Department of Neurology, Rigshospitalet Glostrup from February 01, 2020 to September 01, 2020.

### Headache and Accompanying Symptoms

Immediately before oral glibenclamide or placebo administration, and every 10 min after the administration, we asked participants specifically about the presence of headache, nausea, photophobia, and phonophobia. The features of headache including intensity, location, throbbing/pressing and aggravation by activity were recorded using a standardized questionnaire. Headache intensity was recorded on a numerical rating scale (NRS 0–10) rating pain from none (NRS 0) to maximum imaginable (NRS 10). The participants completed the headache questionnaires hourly until 10 h (waking hours) after discharge from the clinic (Figure 2). If the symptoms fulfilled ICHD-3 beta criteria C and D for migraine without aura (Vincent and Wang, 2018), they were characterized as migraine-like attacks.

### Glucose Infusion

Plasma glucose concentrations were monitored during a 20 min baseline period before the administration of oral glibenclamide/placebo. After the start, and when initial fasting glycaemia had declined by 10%, blood glucose concentrations were clamped around this level (4–7 mmol/L) by 20% glucose infusion. Glucose infusion rates (GIRs) is based on a previous study (Ampudia-Blasco et al., 1994). Infusion rates (IRs) necessary to maintain stable blood glucose after drug intake were registered and adjusted throughout the ensuing 240 min. The following standard formula was used to calculate the IR taking the participants weight into account and ignoring factors such as gender, age and basal metabolic rate:

$$\mathrm{Infusion}\,\mathrm{rate}\,\left(\frac{\text{ml}}{\text{hr}}\right)\,\frac{\text{GIR}\,\frac{\text{mg}}{\mathrm{kg}\times \min}\times \mathrm{weight}\,\left(\text{kg}\right)\times 60\,\frac{\text{min}}{\text{hr}}}{\text{concentration}\,\frac{\text{g}}{100\,m\,l}\times 1,000\,\frac{\text{mg}}{\text{g}}/100}$$

Twenty-nine blood samples were obtained for the determination of glucose during the experiment period. Blood samples were drawn at 5 min intervals between 30 and 90 min, and at 10 min intervals thereafter. The venous blood samples were drawn from the intravenous catheter using a blood gas aspirator (Radiometer, SafePICO, self-filling blood gas syringe) and the blood glucose concentrations were determined with a blood gas analyzer (Radiometer, ABL90 FLEX).

### Statistics and Data Analysis

Baseline was defined as *T*_0_ before the start of oral glibenclamide or placebo administration. For glucose measurement, the baseline was defined as *T*_–__20 *to* 0_ before the start. Calculation of sample size was based on previous similar studies (Vollesen et al., 2019; Ghanizada et al., 2020b). We aimed to detect a 50% difference at 5% significance with 90% power between glibenclamide compared to placebo. We estimated that 17 participants are needed, and we included 20 subjects to ensure power. Headache intensity scores are presented as median (range). The primary endpoints were the difference in incidence of headache and the difference in area under the curve (AUC) for headache intensity scores between two experimental days: glibenclamide-CGRP day (oral glibenclamide followed by infusion of CGRP) versus placebo-CGRP day (oral placebo followed by infusion of CGRP). The secondary endpoints included: the difference in AUC for V_*MCA*_, STA, RA, HR, MAP, and facial skin blood flow between two experimental days. We used the trapezoidal rule to calculate AUC to analyse the differences in response between glibenclamide and placebo. We used Wilcoxon signed rank test to analyse headache intensity scores, paired two-way *t*-test to analyse other variables, and Mann–Whitney *U* test and independent *t*-test to assess period and carry-over effects for all variables. Binary categorical data including the incidence of headache, accompanying symptoms and adverse events were analyzed with McNemar’s test. All analyses were performed with SPSS Statistics version 23 for Windows and a *P*-value < 0.05 was considered as the level of significance.

### Data Availability

The data supporting the findings in the present study are available from the corresponding author, upon reasonable request.

## Results

Twenty healthy volunteers (11 women and 9 men) completed both days of the study. The mean age was 23 years (range 18–27) and mean bodyweight was 72 kg (range 51–94, body mass index range 18–25). There was no difference between the right and left-sided MCA, and therefore the average of both sides was used. There was no carry-over or period effect for values of headache, HR, MAP, V_*MCA*_, STA, or RA.

### Headache and Associated Symptoms

**FIGURE 3:**
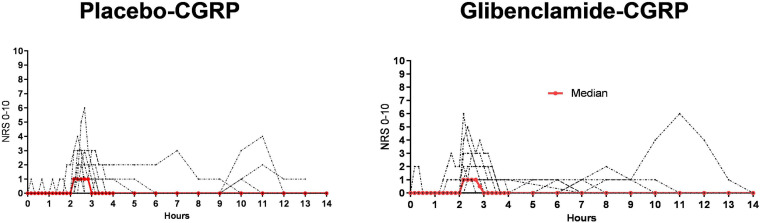
Individual (black lines) and median (red line) headache intensity after glibenclamide and placebo, *n* = 20. All participants received CGRP-infusion at 120 min. On glibenclamide-CGRP day 14 reported headache and on placebo-CGRP day 19 participants reported headache. We found no difference in headache incidence and intensity between glibenclamide-CGRP and placebo-CGRP day (*P* = 0.06).

Fourteen participants reported headache on glibenclamide-CGRP day (14/20, 70%) compared to 19 on placebo-CGRP day (19/20, 95%; *P* = 0.06). The median peak headache intensity score was 1 (range 1–8) (Figure 3) and the median time to peak headache score was 140 min (range 10 min–9 h) on both study days (Table 1). Median headache duration was 105 min on glibenclamide-CGRP day and 95 min on placebo-CGRP day. We found no difference in the AUC_0__–__14 *h*_ for headache intensity after glibenclamide (5.08 ± 7.4) compared with placebo (6.18 ± 10.5; *P* = 0.69). Three participants reported migraine-like attacks after glibenclamide compared with two after placebo (Table 1).

**TABLE 1:** Clinical characteristics of headache and associated symptoms in healthy volunteers after glibenclamide and placebo (0–14 h observation period).

**Participants**	**Peak headache (duration of headache)**	**Headache characteristics**	**Associated symptoms**	**Migraine-like attacks (onset)**	**Treatment (time)/efficacy**
1					
Glibenclamide-CGRP	None				
Placebo-CGRP	140 min (40 min)	Bilat/1/press/−	− / − / −	no	None
2					
Glibenclamide-CGRP	130 min (420 min)	Bilat/3/press/+	− / − /−	no	None
Placebo-CGRP	150 min (310 min)	Bilat/2/press/−	− / − /−	no	None
3					
Glibenclamide-CGRP	140 min (420 min)	Bilat/2/press+throb/+	− / − /−	no	Paracetamol 1 g (9 h/yes)
Placebo-CGRP	130 min (440 min)	Bilat/3/press+throb/+	− / − /−	no	Paracetamol 1 g (7 h/yes)
4					
Glibenclamide-CGRP	140 min (80 min)	Bilat/1/press/−	+ / − /−	no	None
Placebo-CGRP	130 min (50 min)	Bilat/1/press+throb/−	+ / − /−	no	None
5					
Glibenclamide-CGRP	130 min (250 min)	Bilat/6/throb/+	+/ − /−	Yes	None
Placebo-CGRP	10 min (90 min)	Bilat/1/throb+ press/+	−/ − /−	no	None
6					
Glibenclamide-CGRP	None				
Placebo-CGRP	130 min (30 min)	Bilat/1/throb/−	− / − /−	no	None
7					
Glibenclamide-CGRP	none				
Placebo-CGRP	180 min (170 min)	unileft/3/press+throb/−	− / + /−	no	None
8					
Glibenclamide-CGRP	540 min (550 min)	Bilat/6/press+throb/−	− / − /+	no	Paracetamol 1 g (11 h/yes)
Placebo-CGRP	540 min (150 min)	Bilat/4/press+throb/−	− / − /+	no	None
9					
Glibenclamide-CGRP	130 min (50 min)	Bilat/1/press+throb/−	− / − /−	no	None
Placebo-CGRP	130 min (30 min	Bilat/1/throb/−	− / − /−	no	None
10					
Glibenclamide-CGRP	None				
Placebo-CGRP	140 min (20 min)	bilat/3/press/−	− / − /−	no	None
11					
Glibenclamide-CGRP	None				
Placebo-CGRP	None				
12					
Glibenclamide-CGRP	130 min (120 min)	bilat/1/press+throb/+	+/ + /+	Yes	None
Placebo-CGRP	130 min (120 min)	bilat/1/press+throb/+	+/ + /+	Yes	None
13					
Glibenclamide-CGRP	140 min (170 min)	bilat/5/press+throb/−	−/ + /+	Yes	None
Placebo-CGRP	160 min (70 min)	bilat/6/press+throb/−	+/ + /−	Yes	None
14					
Glibenclamide-CGRP	150 min (140 min)	bilat/3/press+throb/+	−/ − /−	no	None
Placebo-CGRP	140 min (100 min)	bilat/4/press+throb/+	−/ + /−	no	None
15					
Glibenclamide-CGRP	130 min (30 min)	bilat/1/press/+	−/ − /−	no	None
Placebo-CGRP	130 min (30 min)	bilat/1/press/−	−/ − /−	no	None
16					
Glibenclamide-CGRP	540 min (440 min)	bilat/8/press/−	+/ − /+	no	None
Placebo-CGRP	150 min (450 min)	bilat/2/press/+	+/ + /−	no	None
17					
Glibenclamide-CGRP	240 min (430 min)	bilat/2/press+throb/−	−/ − /−	no	None
Placebo-CGRP	140 min (20 min)	bilat/1/throb/−	+/ − /−	no	None
18					
Glibenclamide-CGRP	none				
Placebo-CGRP	160 min (410 min)	bilat/2/press+throb/+	− / − /−	no	None
19					
Glibenclamide-CGRP	150 min (90 min)	bilat/1/throb/−	+ / − /+	no	None
Placebo-CGRP	130 min (610 min)	bilat/1/press+throb /+	− / − /−	no	None
20					
Glibenclamide-CGRP	540 min (180 min)	bilat/1/press /−	+/ − /−	no	None
Placebo-CGRP	130 min (70 min)	diffus/1/throb /+	−/ − /−	no	None

### The Hemodynamic Effects

**FIGURE 4:**
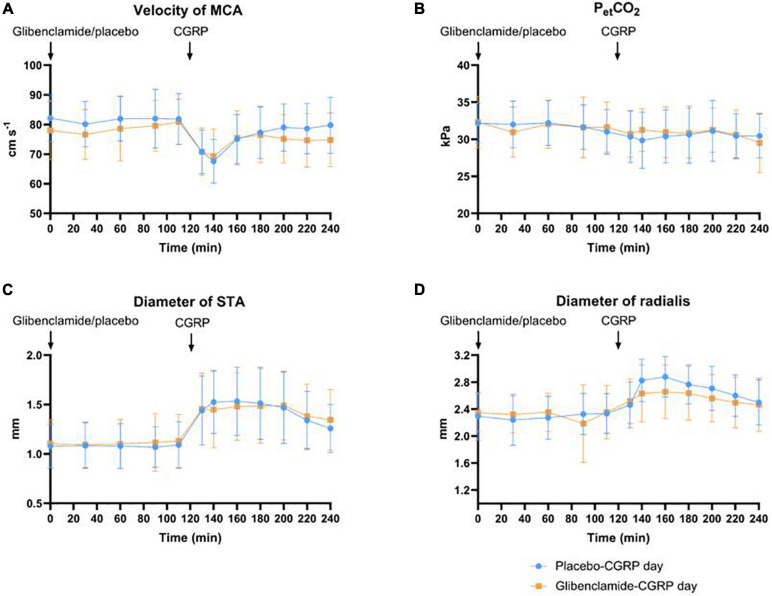
**(A)** Effect of glibenclamide and placebo on middle cerebral artery (MCA) as changes in velocity (cm/s), *n* = 20. There was no difference in AUC_0__–__240 *min*_ for V_*MCA*_ between glibenclamide-CGRP and placebo-CGRP day. **(B)** Changes in end-tidal pCO_2_ were monitored during MCA-measurements. There was no difference in changes in end-tidal PCO_2_ (PetCO_2_) between two experimental days. **(C)** Changes in superficial temporal artery (STA) in diameter (mm). There was no difference in AUC_0__–__240 *min*_ for STA between glibenclamide-CGRP and placebo-CGRP day. **(D)** Changes in radial artery (RA) diameter (mm). No changes in AUC_0__–__240 *min*_ for RA diameter was observed between glibenclamide-CGRP and placebo-CGRP day.

**FIGURE 5:**
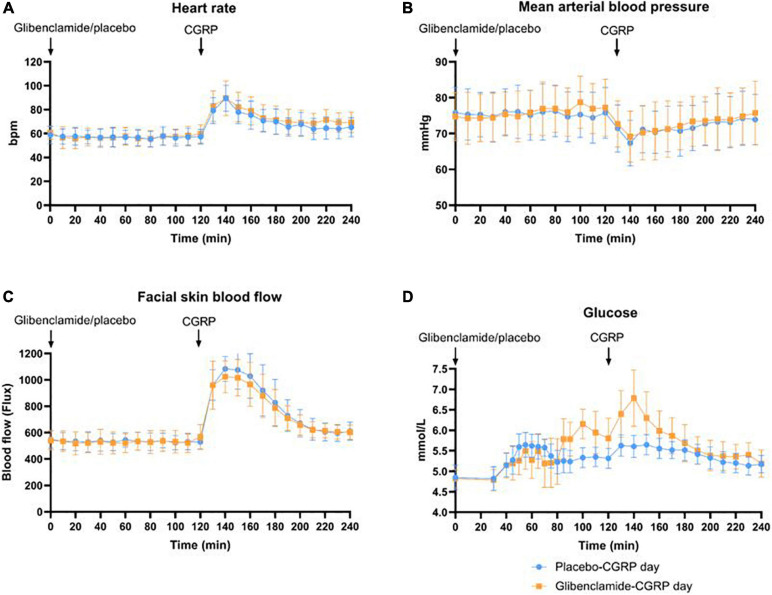
**(A)** Changes in heart rate (bpm) were registered every 10 min. Heart rate did not differ between glibenclamide-CGRP and placebo-CGRP day. **(B)** Changes in mean arterial blood pressure (MAP) in mmHg. MAP did not differ between glibenclamide-CGRP and placebo-CGRP day. **(C)** Facial skin blood flow measured with Laser Speckle as changes in flux. No changes in AUC_0__–__240 *min*_ for facial skin blood flow were observed between glibenclamide-CGRP (157.5 ± 15.4) and placebo-CGRP day (160.8 ± 14.5; *P* = 0.3). **(D)** Blood glucose samples were drawn at 5 min intervals between 30 and 90 min, and at 10 min intervals hereafter. Blood glucose was clamped between 4 and 7 mmol/L.

**FIGURE 6:**
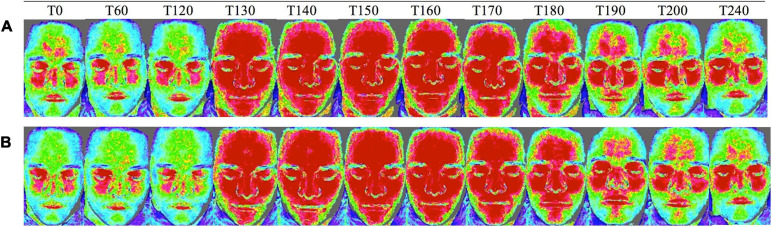
Facial skin blood flow changes measured with laser speckle on **(A)** glibenclamide-CGRP day and **(B)** placebo-CGRP day between *T*_0_ and *T*_240 min_. Blue areas indicate low blood flow, green moderate blood flow, and red high blood flow (Kazmi et al., 2015; Ghanizada et al., 2020a). Upon CGRP infusion at *T*_120 min_ facial skin blood flow increased on both days.

We observed a 21% decrease in V_*MCA*_, 41% dilation of STA and 23% dilation of RA at the end of CGRP infusion (20 min after infusion start) on both days. We found no difference in the AUC_0__–__240 *min*_ for V_*MCA*_ between glibenclamide-CGRP day (83.4 ± 8.5) and placebo-CGRP day (85.5 ± 7.7; *P* = 0.13) (Figures 4A,B). There was no difference in the AUC_0__–__240 *min*_ for STA after glibenclamide (1.4 ± 0.33) compared with placebo (1.4 ± 0.29; *P* = 0.75) and in the AUC_0__–__240 *min*_ for RA after glibenclamide (2.7 ± 0.36) compared with placebo (2.8 ± 0.31; *P* = 0.24) (Figures 4C,D). Pretreatment with glibenclamide caused no change in HR and MAP compared with placebo (Figures 5A,B). Glucose fluctuations due to glibenclamide treatment were avoided by clamping glucose between 4 and 7 mmol/L (Figure 5D). CGRP increased facial skin blood flow on both days (Figures 5C, 6). We found no difference in the AUC_0__–__240 *min*_ for facial skin blood flow after glibenclamide (157.5 ± 15.4) compared with placebo (160.8 ± 14.5; *P* = 0.3).

### Adverse Events

The number of participants who reported flushing, heat sensation and palpitation after glibenclamide did not differ from placebo (*P >* 0.05). Eleven participants experienced fatigue after glibenclamide compared to 4 participants after placebo, and 5 participants experienced thirst after glibenclamide compared to none after placebo (Table 2).

**TABLE 2:** Incidence of adverse events 0–14 h after glibenclamide or placebo.

**Adverse event**	**Glibenclamide-CGRP**	**Placebo-CGRP**
Fatigue	11	4
Flushing	20	20
Warm sensation	19	20
Yawn urge	2	1
Thirst	5	0
Neck stiffness	3	4
Palpitation	18	17

## Discussion

The major outcome of the present study was that K_*ATP*_ channel inhibitor glibenclamide did not affect CGRP-induced headache and vascular changes. The CGRP-induced headache and vascular changes reported in the present study were consistent with previous studies (Petersen et al., 2005b; Hansen et al., 2010; Ashina et al., 2018; Falkenberg et al., 2020). Petersen et al. showed that pretreatment with the CGRP receptor antagonist BIBN4096BS inhibited headache and extracranial vasodilation after intravenous infusion of 1.5μg/min CGRP over 20 min.

*In vivo* studies showed that glibenclamide attenuated CGRP-induced vasodilation in basilar (Kitazono et al., 1993; Faraci and Sobey, 1998), pial (Hong et al., 1996), and dural arteries (Gozalov et al., 2008). The inhibitory effect of glibenclamide on CGRP-induced vasodilation was dose-dependent, and high doses were necessary to observe an effect (Gozalov et al., 2008). Intravenous glibenclamide administration at concentrations of 20–30 mg/kg attenuated CGRP-induced vasodilation (Gozalov et al., 2008), and i.p. injection of 1 and 10 mg/kg glibenclamide attenuated trigeminal pain transmission in rats with spontaneous trigeminal allodynia (Christensen et al., 2020). Since we observed the participants for 5 h, we used 10 mg per oral glibenclamide which is the maximal single dose tested in humans (Williams et al., 1998; Bayerle-Eder et al., 2000; Gori et al., 2005; Hougaard et al., 2020). This corresponds to 0.14 mg/kg, which is substantially lower than doses used in preclinical models. This might explain the insufficient glibenclamide effect to counteract the CGRP-induced vascular changes. However, using equivalent doses in humans would cause severe hypoglycemia and cannot be reasoned.

Interestingly, *in vitro* studies showed that glibenclamide did not affect CGRP-induced dilation of dural arteries and MCA (Kageyama et al., 1993; Gao et al., 1994; Gozalov et al., 2008). In rat coronary arteries, only the combination of glibenclamide and a calcium-activated K^+^ (BK_*Ca*_) channel blocker charybdotoxin could attenuate the effect of CGRP on arteries *in vitro* (Sheykhzade and Berg Nyborg, 2001) suggesting a dual action of K_*ATP*_ and BK_*Ca*_ channels in CGRP-induced vasodilation. The role of BKCa is recently implicated in headache and migraine patophysiology (Al-Karagholi et al., 2020d, 2021b). Possible involvement of other type of potassium channels in CGRP-induced vasodilation cannot be ruled out. Altogether, these data suggest that interspecies differences are likely to contribute to the discrepancy in the findings of glibenclamide on CGRP-induced vascular effects.

Binding of CGRP to the CGRP-receptor complex on vascular smooth muscle cells (VSMCs) (Eftekhari et al., 2013; Edvinsson et al., 2018) mediates an activation of the cyclic adenosine monophosphate-protein kinase A (cAMP-PKA) pathway resulting in potassium efflux through K_*ATP*_ channels, hyperpolarization, and eventually relaxation and vasodilation (Hong et al., 1996; Williams et al., 1998; Ashina et al., 2021). K_*ATP*_ channels consist of different isoforms of sulfonylurea receptor subunits (SUR1, SUR2A, and SUR2B) (Kokoti et al., 2020). SUR1 are expressed in the pancreas (Babenko et al., 2000), the TG and the trigeminal nucleus caudalis (TNC) (Ploug et al., 2012). The SUR2B isoform is of vascular origin and highly expressed in cranial arteries (Ploug et al., 2008a, b, 2012). Glibenclamide is a non-specific SUR subunit blocker shown to have high affinity for SUR1 and low affinity for SUR2B (Inagaki et al., 1996; Stephan et al., 2006). SUR subunits are downstream molecules in CGRP signaling cascades, and whether CGRP receptors and SUR subunits have a cross-reactivity at the same cellular level is yet to be demonstrated. Thus, the results of the present study might be explained by different affinity of glibenclamide and CGRP signaling pathway to SUR isoforms.

In conclusion, we found that pretreatment with glibenclamide did not affect CGRP-induced headache and vascular responses. Our findings could suggest that: (1) the potency of used glibenclamide concentration is too low to result in any measurable effects on CGRP-induced headache and vasodilation, (2) CGRP-induced headache and vascular responses could be initiated by activation of SUR2B K_*ATP*_ channel (Al-Karagholi et al., 2017), and/or (3) interspecies differences might influence the role of K_*ATP*_ channel in CGRP signaling pathway. To clarify the molecular interaction in the CGRP signaling pathway, more studies on specific isoforms of sulfonylurea receptor subunits of K_*ATP*_ channel in humans are needed.

## Data Availability Statement

The raw data supporting the conclusions of this article will be made available by the authors, without undue reservation.

## Ethics Statement

The studies involving human participants were reviewed and approved by the Ethics Committee of the Capital Region of Denmark (H-19065735). The patients/participants provided their written informed consent to participate in this study.

## Author Contributions

All authors listed have made a substantial, direct and intellectual contribution to the work, and approved it for publication.

## Conflict of Interest

The authors declare that the research was conducted in the absence of any commercial or financial relationships that could be construed as a potential conflict of interest.

## Footnotes

1 www.forsogsperson.dk
